# Laser Emission Spectroscopy of Graphene Oxide Deposited on 316 Steel and Ti6Al4V Titanium Alloy Suitable for Orthopedics

**DOI:** 10.3390/ma16072574

**Published:** 2023-03-24

**Authors:** Barbara Nasiłowska, Wojciech Skrzeczanowski, Aneta Bombalska, Zdzisław Bogdanowicz

**Affiliations:** 1 Institute of Optoelectronics, Military University of Technology, gen. S. Kaliskiego 2, 00-908 Warsaw, Poland; 2 Faculty of Mechanical Engineering, Military University of Technology, gen. S. Kaliskiego 2, 00-908 Warsaw, Poland

**Keywords:** LIBS, graphene oxide, stainless steel, Ti6Al4V alloy

## Abstract

This paper presents the results of an analysis of carbon (in the form of graphene oxide) deposited on the surface of threads made from stainless steel 316 and titanium alloy Ti6Al4V used in orthopedics using Laser Induced Breakdown Spectroscopy (LIBS). The aim of the article is to indicate the possibility of using the LIBS spectra for the study of thin layers, including graphene derivatives and other elements. Stratigraphic measurements allowed the detection of differences in the spectra peaks of individual elements, not only in the surface layer itself and in the native material, but also in the intermediate layer connecting the two layers. Due to the clear difference in the outline of the spectrum of graphene oxide and the spectrum of the native material of the samples analyzed, a clear incorporation of carbon atoms into the surface layer was observed. A factor analysis was performed, which confirmed the incorporation of graphene oxide into the surface layer of the native material of the elements examined.

## 1. Introduction

In production technology, construction materials are increasingly being subjected to precise tests in order to diagnose the impacts of the presence of trace elements on the structure and functional parameters of the material [1,2,3,4,5]. In materials science, contamination of a material with trace elements may cause the occurrence of intermetallic phases or carbides which, in turn, may adversely affect the formation of crack initiation. Occasionally, the addition of trace amounts can improve functional properties. However, this small percentage of cases is difficult to diagnose and can represent a reading error limit on various research devices. 

One of the more subtle methods used to detect the presence of changes in the composition of elements of thin films (thickness of several nm and even Å) is Laser Induced Breakdown Spectroscopy (LIBS) [4,6]. This method involves the ablation of material using a laser pulse, resulting in the production of plasma-emitting continuous and linear radiation. The analysis of line radiation emitted by the plasma permits the identification of the elements present in the sample tested. Furthermore, a LIBS analysis makes it possible to obtain qualitative information about the elemental compositions of the samples tested [4,6]. 

The literature describes various applications of the LIBS method to determine the qualitative composition of materials in various states, only in solids, including soils, diatomaceous earths, or uranium oxides, but also in liquids and gases [7,8,9,10].

The detection of pollutants that migrate to shallow groundwater aquifers is presented in [11]. Such studies are particularly important in the context of assessing the sustainability of the storage of natural and artificial water resources. Cantwell et al. [11] presented the results of research on the possibility of real-time monitoring of changes occurring in elemental composition using Scanning Laser Microscopy. These tests were carried out in hard-to-reach places thanks to the flexible design of the probe integrated with the optical fiber. In turn, Guang Pu Xue et al. [12] used a laser with wavelengths of 532 and 1064 nm and energies of 3 and 40 mJ, respectively, for ocean applications (an aqueous solution of CaCl_2_). The results indicated that, compared to a 532 nm laser, a 1064 nm laser can induce plasma in water with a higher emission intensity and longer lifetime. However, the repeatability of the LIBS signal with a 1064 nm laser was lower. Due to the different attenuation factors of laser energy values of 532 and 1064 nm in water, the LIBS signal of the 1064 nm laser decreased significantly over a transmission distance range of 20–50 mm, while the LIBS signal of 532 nm remained the same. 

Lebedev et al. [13] investigated the possibility of using LIBS to study the chemical compositions of different types of nanocarbon structures. Graphene oxide and reduced graphene oxide were chosen as the most attractive representatives of nanocarbon materials. Based on the emission spectra of the plasmas analyzed, they determined the functionalization parameters and the presence of various inorganic pollutants. 

The LIBS method mainly allows the study of the elemental composition, but it is also possible to detect differences in the elemental composition among the surface layers. The uniqueness of this method lies in the use of appropriately selected parameters, which makes it possible to detect intermediate layers that form between the controlled parameters of the surface layer and a specific material. The intermediate layer may indicate the incorporation of the atoms of the surface layer into the base material. Despite the fairly widespread use of Laser Emission Induced Breakdown Spectroscopy, an analysis of the possibility of using graphene oxide identification on surfaces made of steel and nonferrous metals is lacking, in particular regarding threads made from austenitic steel 316 and titanium alloy Ti6Al4V. Therefore, stratigraphic measurements were made deep into the surface layers to determine the possibility of identifying the graphene oxide layer on the surface of the threaded joints.

## 2. Materials and Methods

A graphene oxide deposition process was performed on the surface of screws made from 316 stainless steel (in % of Ni 10.0–13.0; Cr 16.5–18.5; Mn < 2.0; C < 0.03; Si < 1.0; P < 0.045; S < 0.015; N < 0.11; Fe = bal.) and titanium alloy Ti6Al4V (in % of Al 5.50 to 6.75; V 3.50–4.50; Fe < 0.30; About < 0.20; C < 0.08; N < 0.05; Ti = bal.) at the Biomedical Engineering Center of the Institute of Optoelectronics of the Military University of Technology, according to the methodology developed, which included plasma purification followed by the deposition of graphene oxide using centrifugal forces (centrifugal method) and vacuum drying of screws with an embedded graphene layer (patent application). The process of graphene oxide deposition on the screw surfaces is described in [14].

A dispersed suspension of graphene oxide purchased from the Department of Chemical Synthesis and Flake Graphene, Łukasiewicz Research Network—Institute of Electronic Materials Technology, Warsaw, Poland was used in this work. The manufacturing process and structural characteristics are described in [15].

**Figure 1:**
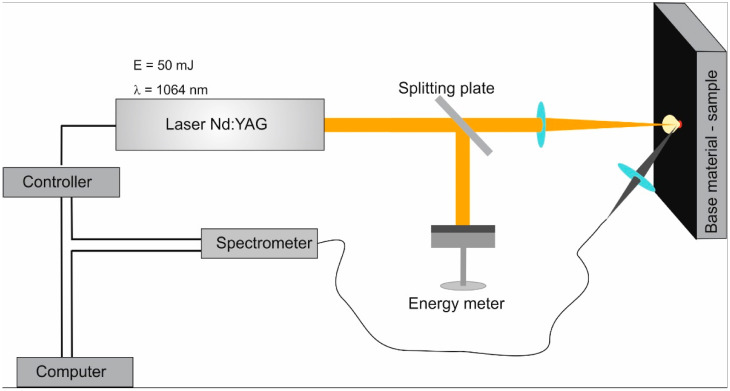
Scheme of the measurement system.

An experiment aimed at determining the chemical composition of a specimen of graphene paper and carbon composite was conducted with Laser Induced Breakdown Spectroscopy (LIBS method). A laser beam was focused on a specimen of the tested material causing its ablation and, subsequently, the heating and ionization of the vapor produced as well as the generation of plasma. The plasma generated in this way was a source of a strong continuum of discrete radiation specific to atoms in the given specimen. The tests were carried out using the experimental setup presented in Figure 1. This experimental geometry (vertical position of the sample) was chosen to minimize the redeposition of particles ripped out of the sample by ablation in former laser shots which, in the stratigraphy experimental mode, makes the results obtained more credible.

To generate the plasma, a pulse laser Nd:YAG (Brio model, produced by Quantel) was used (Bozeman, MT, USA). The tests were conducted with 1064 nm radiation generating 4 ns pulses. The plasma radiation was recorded using a spectrometer, enabling time-resolved measurements. An ESA 4000 echelle spectrometer (LLA Instruments GmbH& Co KG, Berlin, Germany) was used, which allowed the spectra to be detected in time windows of between 20 ns and 16 ms in the 200–800 nm spectral range. The detection part was the Kodak KAF 1001 CCD matrix with the ICCD amplifier embedded in the ESA 4000 device. The spectral resolution of the whole detection system was λ/Δλ~20,000. 

**Figure 2:**
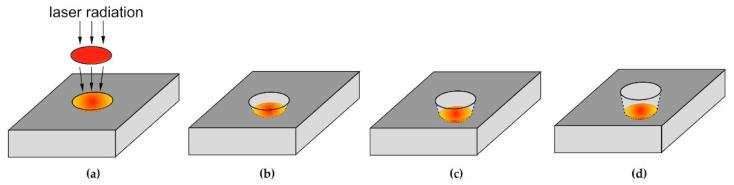
Diagram of stratigraphic measurements made in the LIBS experiment (**a**) initial phase, before laser shot, (**b**–**d**) sample surface with increasing number of laser shots.

The analysis of the stratigraphic spectra consists of making an incremental number of laser shots that are subsequently deposited on the same spot and then recording the number of spectra each created by successive laser shots (Figure 2). 

In the study, we did not analyze the influence of the shape of the thread profile on the test result. We used a 150 mm focus lens, and the lens-target/thread distance was set to 140 mm to avoid an accidental breakdown in the air. This geometry resulted in the focal diameter of the laser spot being equal to approximately 0.3 mm, and it was located on a flat surface between the screw head and the thread profile. In this arrangement, laser fluence in the focal spot on the screw amounted to 0.14 J/mm^2^. At this fluence, the ablation rate, a very important parameter in laser processing, amounted to ~10 nm/pulse for 316 steel and Ti alloy. However, in LIBS experiments, the ablation rate is not a crucial parameter, as it is in the laser processing of solids.

The spectra obtained were subjected to statistical processing using multivariate factor analysis (FA). FA analysis relies on the reduction of many input variables (in this case 10 LIBS spectra), which can be correlated with each other (not always clearly), to a much smaller number of new, uncorrelated variables—so-called factors. The transformation procedure leading to the determination of new variables is constructed so that the first variable contains the largest range of input variability, the next a slightly smaller range, and so on.

It is assumed that, in order to describe the input dataset correctly, the first two factors (main components) should describe more than 70% of the variation (variance) of the input set. The entire FA analysis was performed using the STATISTICA 10 PL program. The statistical analysis was carried out in such a way that a matrix consisting of over 1 million elements constituting appropriately modified LIBS spectra was entered into the STATISTICA 10 PL program. Factor analysis made it possible to classify the input data, which means that, as a result of data processing, measurement points representing individual spectra were obtained in the new coordinate system, and their locations were used to determine the degree of similarity (or identity) in the spectra: when the points representing LIBS spectra, meaning the chemical compositions at the place at which the spectra waere registered, lie closer to each other, they have more similar chemical compositions [16].

Structural studies were carried out using a Quanta 250 FEG scanning electron microscope (Quanta 250 FEG SEM, FEI, Hillsboro, OR, USA).

Raman spectra were recorded with the use of a ThermoScientific Nicolet iS50 Raman spectrometer (ThermoFisher SCIENTIFIC, Waltham, MA, USA). An excitation line of 1064 nm was used, and spectra were collected in a range of 400–2000 cm^−1^ with 300 repetition scans and a laser power of 0.1 W. Screws were placed on the controlled moving table to establish the laser focus. The largest accessible area was near the head of the screw. The spectra were collected from 5 places for each screw. 

## 3. Results

### 3.1. Structural Analysis 

**Figure 3:**
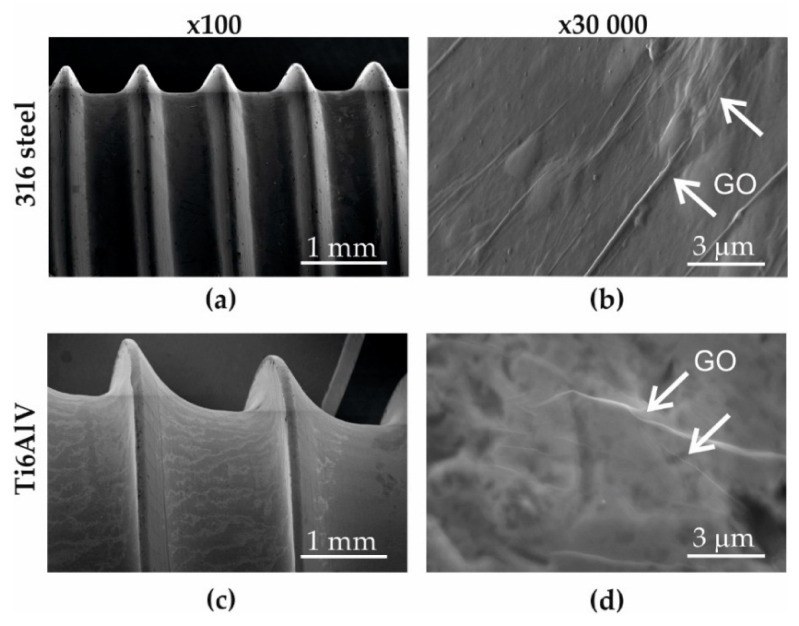
SEM image of the screw surface: (**a**,**b**) stainless steel 316 and (**c**,**d**) alloy Ti6Al4V (the arrows point the GO layers).

Structural studies conducted using a scanning electron microscope showed that the screws made from steel 316 (Figure 3a,b) and titanium alloy Ti6Al4V (Figure 3c,d) were characterized by an almost uniform layer of graphene oxide, not only on the surface but also at the bottom of the geometric notches. No discontinuities, gaps, or fissures that could expose the native material were observed within the layers of superimposed graphene oxide flakes. The thickness of the graphene oxide layer was about 30 nm.

### 3.2. Raman Surface Analysis

**Figure 4:**
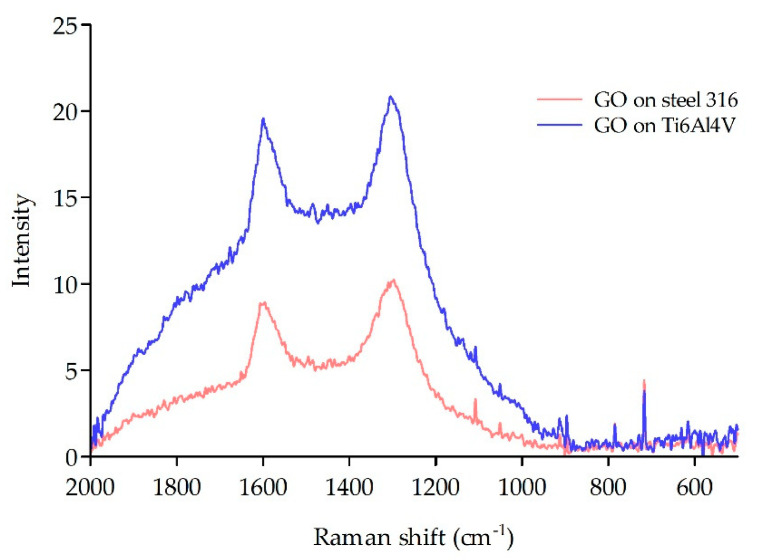
Raman spectrum of screws examined with deposited GO.

Two characteristic bands were observed at 1600 and 1302 cm^−1^ in both groups of samples. The screws made from the Ti6Al4V alloy showed a higher GO content on the surface and higher background scattering. Screws made from stainless steel 316 presented a 30% lower GO content than those made from the titanium alloy Ti6Al4V (Figure 4).

### 3.3. LIBS Analysis

**Figure 5:**
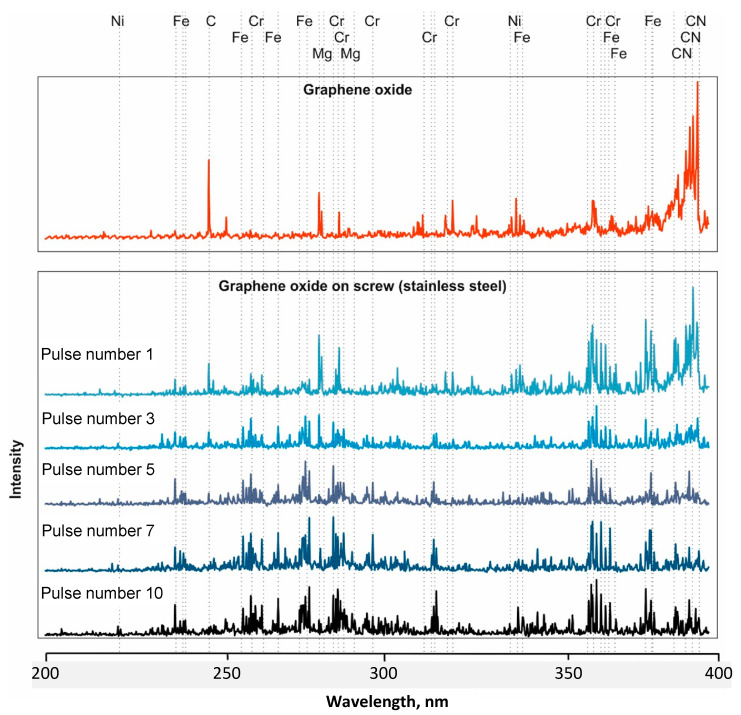
LIBS spectra of graphene oxide deposited on a thread made from steel 316L obtained with a different number of laser beam pulses and related to the spectrum of graphene oxide.

**Figure 6:**
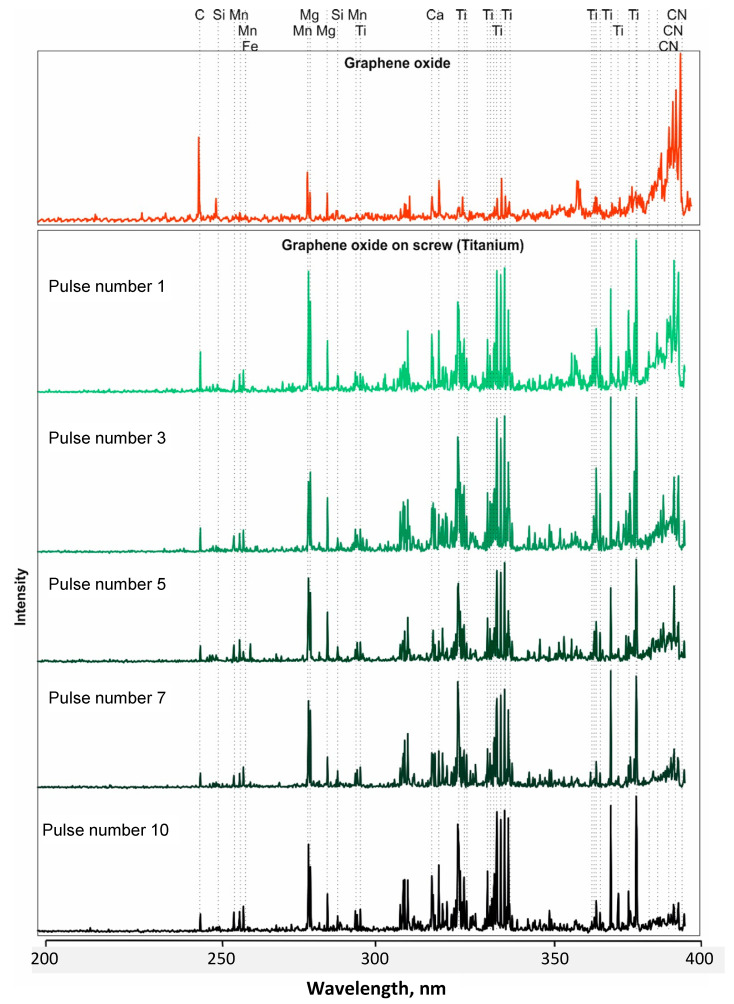
LIBS spectra of graphene oxide deposited on a thread in a titanium alloy Ti6Al4V, obtained with a different number of laser beam pulses and related to the spectrum of graphene oxide.

Selected stratigraphic spectra of graphene oxide (pulse numbers 1, 3, 5, 7, and 10) deposited on the surface of screws made from austenitic steel 316 (Figure 5) and titanium alloy Ti6Al4V (Figure 6) are presented below. These spectra were related to the spectrum of graphene oxide so that changes in the peaks depending on the next number in the laser pulse were visible. The spectrum obtained from graphene oxide was dominated by C and CN compounds, and admixtures were also visible, including elements such as Mg, Cr, and Ni. The strongest C peaks and CN bands were observed at only 1 pulse of the laser beam on a screw made from steel 316 (Figure 5). In Figure 5, we present a short-wavelength part of the LIBS spectra, namely in the 200–400 nm spectral interval. The entire spectral range in which spectra were recorded covered the 200–800 nm range, but the most visible changes in the spectra, i.e., intensities of carbon/graphene peaks or CN bands as well Fe and Ti lines, were easily found exactly in the 200–400 nm interval. 

In subsequent pulses, peaks from the parent material containing Cr, Ni, and Fe from the magnesium admixture prevailed. Studies have shown that graphene oxide is more embedded in a thread made from titanium alloy Ti6Al4V than in one made from austenitic steel 316. At the 10th pulse acting on the surface of the titanium alloy Ti6Al4V, faint peaks from C and the CN compound were still visible (Figure 6). The analysis of graphene oxide emission spectra showed oscillation–rotational transitions in the CN molecules.

**Figure 7:**
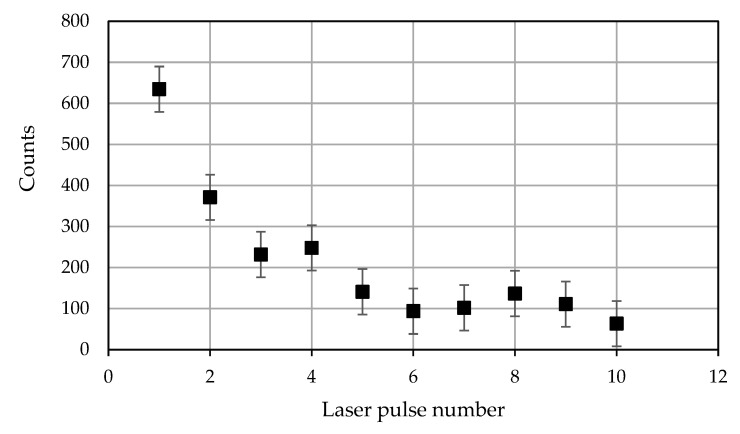
Peak intensity of the carbon line C I 247 nm for the 316L steel sample.

**Figure 8:**
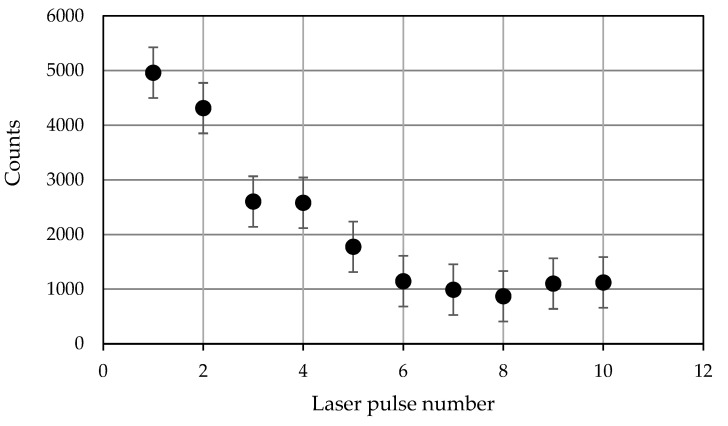
Peak intensity of the carbon line C I 247 nm for the Ti6Al4V alloy.

Figure 7 and Figure 8 illustrate changes in the intensity of carbon from deposited graphene oxide as a function of the laser shot number, i.e., deep into the surface layer. The analysis of the peak intensity showed that the strongest carbon peaks were observed in the first measurements for both a sample made from 316 stainless steel and one made from titanium alloy Ti6Al4V. Studies have shown that the highest peaks were observed for samples made from Ti6Al4V alloy, even for further laser shots. In addition, Figure 7 and Figure 8 show measurement errors resulting from fluctuations in the energy of the laser beam and signal reading by the detector. 

**Figure 9:**
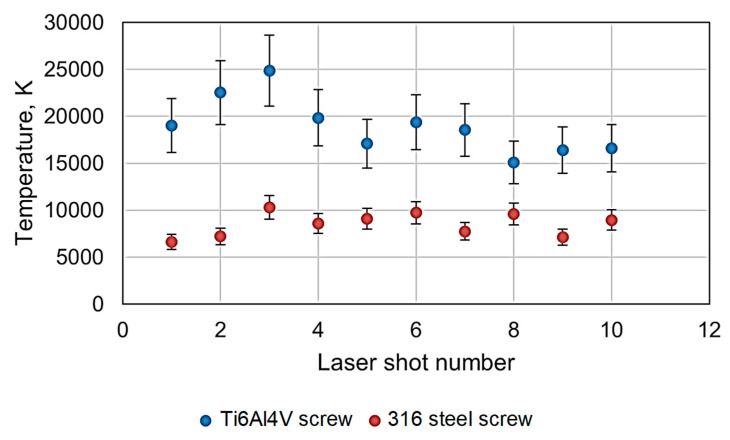
Plasma temperatures for subsequent shots in the stratigraphy experiment.

Although the peak intensities varied significantly during stratigraphy experiments, the plasma temperatures did not change very much, especially for the 316 steel screw. This is shown in Figure 9 in which temperatures derived from Boltzmann plots are presented for the 316 steel and Ti6Al4V alloy screws. It is evident that the temperature for the Ti alloy screw was more than twice the value measured for the 316 steel screw (18,966 K vs. 8960 K—average values). This is due to the lower heat conductivity of titanium compared with steel (~6 W/m∙K for Ti6Al4V [17] vs. ~18–24 for 316 steel [18]). On the other hand, it has a higher absorption coefficient for 1064 nm with respect to steel (~65% for Ti vs. 30% for iron [19,20]). 

The lower heat conductivity of Ti leads to poorer heat dissipation in the area of laser–matter interaction compared with the steel sample. This, in turn, caused a local temperature rise in the laser spot on the target, the location of plasma creation, which should be higher for the Ti than for the steel screws. On the other hand, as has been stated in a number of studies, for example, in [21,22,23], for targets with elevated temperatures, the plasma temperatures measured were higher.

In temperature measurement, experimental errors were determined in the following way: for each sample, a standard deviation from 10 temperature measurements was determined, and then, a relative standard deviation was determined. Error bars are related to the measured temperature values for each depth. In our opinion, the temperature courses indicate relatively stable and similar thermodynamic conditions for the stratigraphy measurements. 

**Figure 10:**
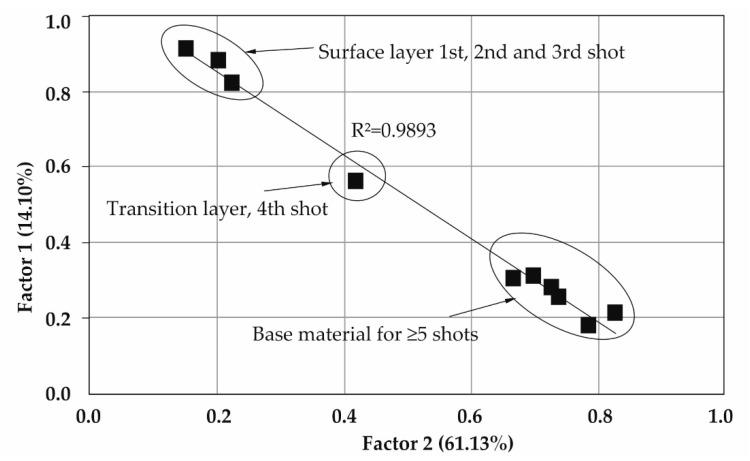
Factorial analysis of the LIBS spectra of a 316 stainless steel sample.

**Figure 11:**
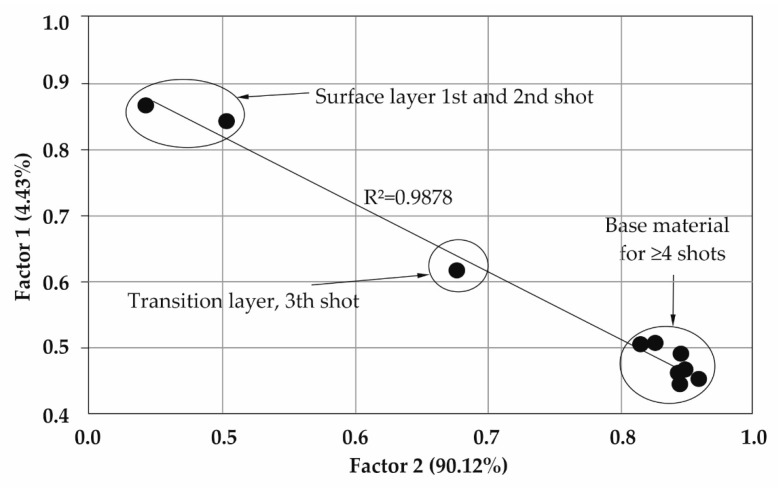
Factorial analysis of the LIBS spectra of a Ti6Al4V alloy sample.

A factorial analysis of the LIBS spectra recorded for GO deposited on stainless steel 316 and alloy Ti6Al4V is shown in Figure 10 and Figure 11. In order to determine the incorporation of carbon (derived from deposited graphene oxide) into the surface layers, two groups of factors were used. Factor 1 was related to the depth in the sample increasing with the number of laser shots, while factor 2 represented the carbon/graphene density.

In factorial space, spectra are arranged in a straight line. The closeness of the points on the graph (within a layer) indicates that the chemical compositions are very similar. 

For both samples tested, the transition layer point marked in Figure 10 and Figure 11 was observed. The thicker layer of carbon derived from deposited graphene oxide was located on the surface of the 316 steel sample.

## 4. Discussion

An analysis of changes in the elemental composition of graphene oxide using Laser Induced Breakdown Spectroscopy (LIBS) was carried out on the surfaces of threads made from 316 stainless steel and titanium alloy Ti6Al4V. Due to the curvature of the surface, the thickness of the layer was not examined, but the main goal of the study was to determine the appearance of an intermediate layer between strong C and CN peaks derived from graphene oxide and the base material using a factor analysis. 

Changes in the presence of spectra peaks characteristic of individual elements were observed (Figure 5 and Figure 6). In the first measurement, the contents of C and the CN molecule were the highest, and then successive changes occurred as the number of pulses increased. The research showed that the intensity of the carbon derived from graphene oxide deposited on the surface of the tested threads made from steel 316 and titanium alloy Ti6Al4V, together with the depth (laser pulse number), significantly decreased and stabilized at a level of >5 laser pulses.

The formation of an intermediate layer between the first layer and the peaks derived from the base material indicates the correct embedding of GO on the materials tested according to the technology developed (Figure 10 and Figure 11). 

The appearance of the intermediate layer was confirmed, to some extent, by the course of changes in the intensity of the signals of carbon C I 247 nm, shown by the stratigraphic data presented in Figure 7 and Figure 8. This may be due to surface activation using plasma [24,25]. In [24,25], it was shown that the interaction of plasma with the surface tested improves the hydrophilicity of a dispersed aqueous suspension containing graphene oxide flakes and allows the free flow of the suspension in microholes, despite the roughness and development of the surface.

## 5. Conclusions

The use of the LIBS method allowed surface layers to be characterized in samples made from steel 316 and titanium alloy Ti6Al4V. The results of emission spectroscopy with laser excitation developed using a factor analysis allowed the following layers to be identified.

- I layer—C dominant
- II Layer—transitional
- III Layer—dominance of the elements of the base material.

The curvature of the surface did not affect the detection of the abovementioned layers.

The results obtained by emission spectroscopy with LIBS laser excitation indicate the possibility of using the LIBS method to analyze the incorporation of GO into the base material. It was observed that the GO was built into the surface of a thread made from titanium alloy Ti6Al4V and steel 316.

## 6. Patents

The processing of elements presented in this work consisted of plasma cleaning and surface activation, the application of graphene oxide, vacuum drying, and mechanical shot peening. This is known as hybrid graphene treatment and is presented in patent application P. 438715 [14].

## Data Availability

Not applicable.
